# A systematic review of multi-level stigma interventions: state of the science and future directions

**DOI:** 10.1186/s12916-018-1244-y

**Published:** 2019-02-15

**Authors:** Deepa Rao, Ahmed Elshafei, Minh Nguyen, Mark L. Hatzenbuehler, Sarah Frey, Vivian F. Go

**Affiliations:** 1 Department of Global Health, Department of Psychiatry and Behavioral Sciences, University of Washington, Campus Mailbox 357965, Harris Hydraulics Building, 1705 NE Pacific Street, Seattle, WA 98195-7175 USA; 2 Department of Global Health, University of Washington, Seattle, WA USA; 3 Department of Health Behavior, Gillings School of Public Health, University of North Carolina, Chapel Hill, NC USA; 4 Department of Sociomedical Sciences, Mailman School of Public Health, Columbia University, New York, NY USA

**Keywords:** Stigma, Multi-level interventions, Low- and middle-income countries

## Abstract

**Background:**

Researchers have long recognized that stigma is a global, multi-level phenomenon requiring intervention approaches that target multiple levels including individual, interpersonal, community, and structural levels. While existing interventions have produced modest reductions in stigma, their full reach and impact remain limited by a nearly exclusive focus targeting only one level of analysis.

**Methods:**

We conducted the first systematic review of original research on multi-level stigma-reduction interventions. We used the following eligibility criteria for inclusion: (1) peer-reviewed, (2) contained original research, (3) published prior to initiation of search on November 30, 2017, (4) evaluated interventions that operated on more than one level, and (5) examined stigma as an outcome. We stratified and analyzed articles by several domains, including whether the research was conducted in a low-, middle-, or high-income country.

**Results:**

Twenty-four articles met the inclusion criteria. The articles included a range of countries (low, middle, and high income), stigmatized conditions/populations (e.g., HIV, mental health, leprosy), intervention targets (e.g., people living with a stigmatized condition, health care workers, family, and community members), and stigma reduction strategies (e.g., contact, social marketing, counseling, faith, problem solving), with most using education-based approaches. A total of 12 (50%) articles examined community-level interventions alongside interpersonal and/or intrapersonal levels, but only 1 (4%) combined a structural-level intervention with another level. Of the 24 studies, only 6 (25%) were randomized controlled trials. While most studies (17 of 24) reported statistically significant declines in at least one measure of stigma, fewer than half reported measures of practical significance (i.e., effect size); those that were reported varied widely in magnitude and were typically in the small-to-moderate range.

**Conclusions:**

While there has been progress over the past decade in the development and evaluation of multi-level stigma interventions, much work remains to strengthen and expand this approach. We highlight several opportunities for new research and program development.

## Background

Stigma can aggravate disease processes and add numerous socioeconomic, psychosocial, and health burdens on people who hold marginalized identities or statuses, including reduced educational attainment, exposure to psychosocial stressors, and challenges in accessing healthcare [1]. Behavioral scientists have studied the severe negative consequences of stigma for individuals coping with various health conditions and have learned that stigma can deter individuals from optimally engaging in treatment for their condition, which has serious impacts on morbidity and mortality [2]. Strikingly, when disease morbidity and mortality are low but the condition is highly stigmatized, the burden of stigma may exceed the burden of the disease in its impact on social, emotional, and work functioning, thus negatively affecting the overall quality of life [3]. Researchers have long recognized that stigma operates on intrapersonal, interpersonal, organizational, and structural levels, and as such, stigma is conceptualized as an inherently multi-level phenomenon [1]. The multi-level nature of stigma renders the development of stigma interventions particularly challenging, in part because addressing multiple levels through research is more complex, requires more resources, and may be more burdensome to participants than single-level interventions. However, for research teams willing to take on the task of addressing multiple levels, the impacts on stigma reduction efforts can be farther reaching, more synergistic, and more holistic than single-level interventions [4].

Two previous papers have reviewed the literature on stigma reduction interventions. In Heijnders and Van Der Meij’s 2006 review [5], consistent with the multi-level approach to stigma [1], the authors identified five levels of examination and mapped strategies and target populations directly onto each level. First, at the *intrapersonal* level, the focus of interventions is on characteristics of the individuals living with a stigmatized condition, and strategies involve self-help, counseling, and treatment. Second, at the *interpersonal* level, the intervention is focused on the enhancement of care and support in the stigmatized persons’ local environment. Third, at the *community* level, the focus is on reducing stigmatizing attitudes and behaviors in (non-stigmatized) community groups using strategies such as education, contact, and advocacy. Heijnders and Van Der Meij define contact as any interactions between the public and the affected person for the purpose of reducing stigma [5]. Fourth, at the *organizational/institutional* level, interventions focus on reducing stigma in an organization or institution, and strategies include training programs and institutional policies. Fifth, at the *governmental/structural* level, interventions focus on establishing and enforcing legal, policy, and rights-based structures.

In 2014, Cook and colleagues [6] conducted a narrative review that similarly considered multiple levels in which stigma interventions can operate as part of an ecological system [7], but focused on only three levels: intrapersonal, interpersonal, and structural. Cook et al.’s definitions of these levels differed slightly from those of Heijnders and Van Der Meij’s and were more flexible, in that one strategy, such as education, could operate on multiple levels. The authors’ primary purpose was to describe how each strategy operates on multiple levels, while targeting both stigmatized and non-stigmatized populations.

Although neither review was systematic, both challenged investigators to build and evaluate multi-level stigma reduction interventions. In Heijnders and Van Der Meij’s review [5], while all of the strategies reviewed had the potential to operate on multiple levels, the authors reviewed studies that evaluated stigma reduction strategies at a single level of analysis. In their conclusion, they called for researchers to combine multiple strategies to target multiple levels. Cook and colleagues [6] conducted an updated narrative review of stigma interventions and analyzed these studies for cascading impacts across multiple levels. The authors determined that studies examining cascading effects across levels were rare, concluding that stigma reduction interventions that examine effects across levels were urgently needed. While these two prior reviews pointed out important lacuna in the literature on stigma interventions, our study addresses another knowledge gap by conducting the first systematic review of multi-level stigma interventions. We describe the country of origin of research studies, depict the design and participants of each multi-level stigma intervention, discuss the strategies and outcomes used by these interventions, and highlight opportunities for new research and program development.

## Methods

We conducted this review in accordance with the Preferred Reporting Items for Systematic Reviews (PRISMA) guidelines [8]. We included studies that focused on stigma reduction interventions operating on multiple levels, both within and outside of the USA. The overall purpose of our review was descriptive, rather than evaluative. Thus, for each study, we provide a basic indication of effectiveness in reducing stigma, but a detailed evaluation of study effectiveness was beyond the scope of this review.

We used Heijnders and Van Der Meij’s categories for the levels of the ecological system (i.e., intrapersonal, interpersonal, community, organizational/institutional, governmental/structural) [5]. However, we expanded our organization of these predefined strategies (e.g., education, contact) such that they could map onto multiple levels, which Heijnders and Van Der Meij did not do in their analysis. For example, if one target of an intervention was to improve attitudes held, whether by the stigmatized or the non-stigmatized, we categorized this focus at the *intrapersonal* level. If an intervention’s target was to improve interactions between people with stigmatized conditions and other stakeholders (e.g., caregivers, healthcare workers), we categorized this focus at the *interpersonal* level. If the (non-stigmatized) public was targeted, we identified the *community* level as the focus. If an organization was targeted, we identified the *organizational/institutional* level as the focus. If a policy or administrative structure was targeted, we identified *governmental/structural* level as the focus.

### Search terms

For our systematic review, we input search terms into six electronic database sources (PubMed, Embase, CINAHL, Global Health, Scopus, and PsychINFO). We looked for all papers with the term “stigma” plus at least one of the following terms: “intervention,” “program,” “programme,” or “policy” in either the title or abstract. We used the Covidence database [9] to extract and organize information from articles. Because of our focus on health-related stigmas, we used primarily health-based databases in our search.

### Inclusion criteria

We used the following eligibility criteria for inclusion: (1) peer-reviewed, (2) contained original research, (3) published prior to initiation of search on November 30, 2017, (4) evaluated interventions that operate on more than one level, as defined above, and (5) examined stigma as an outcome.

### Exclusion criteria

We excluded protocol papers, papers published in languages other than English, abstracts without full texts available, non-peer reviewed articles, and solely qualitative studies.

### Data extraction

**Fig. 1:**
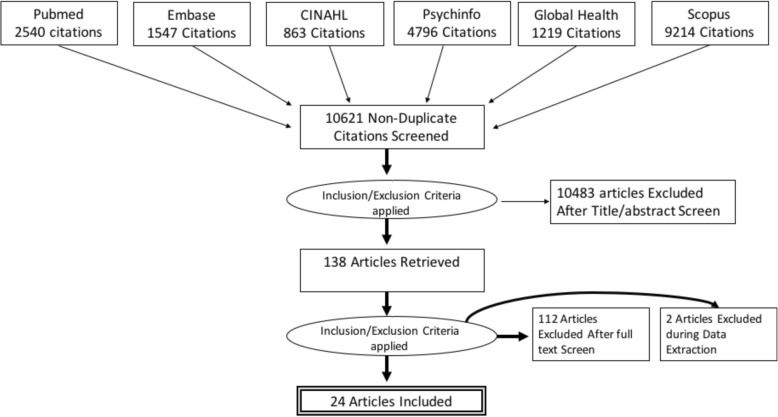
Flow of article inclusion and exclusion from review

After identifying a list of all relevant records and removing duplicates, 10,621 titles remained for title, abstract, and full-text screening. The abstract/title review and subsequent full-text review of the selected studies were conducted independently by two investigators (AE and MN), who had approximately 99% agreement, disagreeing on only 39 of 10,621 articles. Discrepancies were resolved over discussions with two additional investigators (DR and VG). The investigators retained 138 articles after abstract screening and 24 articles after full-text screening based on the inclusion/exclusion criteria above. One hundred and fourteen articles were excluded during full-text screening because we found the articles met exclusion criteria only after reviewing the full text. This process is depicted in Fig. 1.

### Data analysis

We used content analysis [10] to organize the selected qualifying studies. DR and SF independently coded each article. We read through each article and systematically created and collapsed categories. When SF and DR encountered discrepancies, the codes were discussed and adjusted by consensus and the levels, as presented above. The themes identified from the articles included the following: condition/population studied (e.g., HIV, mental health, substance use, leprosy, diabetes, epilepsy, orphaned and vulnerable children), intervention targets (e.g., people living with a condition, health care workers, caregivers/family members, community members), level of intervention targeted, country that served as the setting for the study, and stigma reduction strategies used in the interventions (e.g., education, contact, social marketing, counseling, faith, problem solving). We also coded articles for information on the intensity of the strategies used (e.g., duration, number of sessions) and whether the studies used validated stigma measures. We examined effectiveness using a simple, parsimonious approach, categorizing findings in terms of statistical significance (at least 1 measure of stigma used showed statistically significant reduction) or statistical non-significance (no statistical significance found or no inferential statistics used). We provided confidence intervals when given in the articles, and effect sizes if given or if enough information was given to calculate effect sizes in the articles.

## Results

Overall, six of the 24 studies were randomized controlled trials (two used individual randomization [11, 12] and four used cluster randomization [13–16]) (Table 1). Eighteen studies did not randomize or use a control group and thus were not considered randomized controlled trials (RCTs). Most studies reported on pilot trials of interventions. Of the studies reviewed that did use RCT designs, four used cluster randomization. All studies used convenience samples rather than population-based sampling.

**Table 1:** Multi-level stigma interventions

Author	Title	Country	Condition	Intervention targets	Strategy/intensity	Levels	Stigma measures	Effectiveness
1. Batey, Whitfield, Mulla, Stringer, Durojaiye, McCormick, Turan, Nyblade, Kempf, Turan, 2016 [27]	Adaptation and Implementation of an Intervention to Reduce HIV-Related Stigma Among Healthcare Workers in the United States: Piloting of the FRESH Workshop	US	HIV	17 health care workers (HCW) 19 People living with HIV (PLWH)	Education, contact, coping/counseling; 1.5 days	Intrapersonal Interpersonal	Multi-country Validated Measures for HCW and PLWH at pre- and post-intervention	Non-significant; CI Not Given; ES Not Given
2. Bhana, Mellins, Petersen, Alicea, Myeza, Holst, Abrams, John, Chhagan, Nestadt, Leu, McKay, 2014 [28]	The VUKA family program: Piloting a family-based psychosocial intervention to promote health and mental health among HIV infected early adolescents in South Africa	South Africa	HIV	65 adolescents and their caregivers/family	Education, problem solving, communication; 10 sessions over 3 months	Intrapersonal Interpersonal	US-Validated Measure of Epilepsy Stigma for Adolescents at pre-, 2 weeks post-intervention, 3 months post-intervention	Non-significant; CI not given; ES not given
3. Bogart, Hemmesch 2016 [29]	Benefits of support conferences for parents of and people with Moebius syndrome	US	Moebius	47 People with (PW) Moebius and 48 caregivers/family	Education, contact, social support; 3-day conference	Intrapersonal Interpersonal	US-Validated Measure of Visible Differences at pre- and post- conference attendance	Non-significant; CI not given; ES *d* = 0.5 and *r* = − 0.15
4. Brown 2009 [18]	Faith-based mental health education: A service-learning opportunity for nursing students	US	Mental illness (MI)	55 nursing students, 38 community members	Education; 90-min workshop	Interpersonal Community	Multi-Country Validated Measure of Mental Illness Stigma at pre- and post-workshop	Significant; CI not given; ES *d* = 0.4
5. Chidrawi, Greeff, Termane, Doak 2016 [30]	HIV stigma experiences and stigmatisation before and after an intervention	South Africa	HIV	18 PLWH, 60 caregivers/family	Education, contact, problem solving; 5-month intervention with workshops and group projects	Intrapersonal, Interpersonal	Multi-country Validated HIV Stigma Measure at pre-intervention and quarterly for 1 year	Significant; CI not given; ES *d* = 0.11 (caregivers) *d* = 2.51 (PLWH)
6. Dadun, Van Brakel, Peters, Lusli, Zweekhorst, Bunders, Irwanto 2017 [14]	Impact of socio-economic development, contact and peer counselling on stigma against persons affected by leprosy in Cirebon, Indonesia - a randomized controlled trial	Indonesia	Leprosy	237 PW leprosy, 213 and 375 community	Contact Socio-economic Coping/counseling; 5 counseling sessions; 2 years with community	Intrapersonal Interpersonal Community	Multi-country Validated Leprosy Stigma Scale at pre- and post-intervention	Significant; CI not given; ES *d* = 1.57
7. Hawke, Michalak, Maxwell, Parikh 2014 [31]	Reducing stigma toward people with bipolar disorder: impact of a filmed theatrical intervention based on a personal narrative	Canada	MI	48 PW MI and caregivers/family, 29 community, 60 HCW	Education contact (filmed); 50 min	Intrapersonal Interpersonal Community	Multi-country Validated Stigma/Social Distance of MI Scales at pre-, post- and 1-month post- intervention	Significant; CI not given; ES Eta squared 0.32
8. Henderson, Corker, Lewis-Holmes, Hamilton, Flach, Rose, Williams, Pinfold, Thornicroft 2012 [32]	England’s Time to Change antistigma campaign: One-year outcomes of service user–rated experiences of discrimination	UK	MI	1584 community	Social marketing; 12 months	Interpersonal Community	Multi-country Validated Stigma of MI Scale	Significant; CI not given; ES not given
9. Jürgensen, Sandoy, Michelo, Fylkesnes 2013 [13]	Effects of home-based Voluntary Counselling and Testing on HIV-related stigma: Findings from a cluster-randomized trial in Zambia	Zambia	HIV	1694 community	Voluntary counseling and testing over 2 months	Interpersonal Community	Multi-country Validated HIV Stigma Measure at pre- and 6 months post-intervention	Significant; CI (− 1.08, − 0.054); ES *R*^2^ = 0.02
10. Li, Wu, Liang, Lin, Zhang, Guo, Rou, Li 2013 [15]	An intervention targeting service providers and clients for methadone maintenance treatment in China: A cluster-randomized trial	China	Substance abuse	41 HCW, 179 people using heroin on methadone	Education (3 group sessions), motivational interviewing (2 sessions)	Intrapersonal Institutional	Chinese Validated Perceived Stigma of Addictions Care at pre-, 3, 6, 9 months post intervention.	Non-significant; CI 3 months (− 3.36, 3.01); 6 months (− 6.96, 1.21); 9 months (− 6.39, 2.66); ES Not Given
11. Lusli, Peters, van Brakel, Zweekhorst, Iancu, Bunders, Irwanto, Regeer 2016 [16]	The impact of a rights-based counseling intervention to reduce stigma in people affected by leprosy in Indonesia	Indonesia	Leprosy	124 PW leprosy	Coping/counseling, 5 sessions	Intrapersonal Interpersonal	Adapted from a Multi-Country Validated Measure of HIV Stigma at pre- and post-intervention	Significant; CI not given; ES not given
12. Maulik, Kallakuri, Devarapalli, Vadlamani, Jha, Patel 2017 [33]	Increasing use of mental health services in remote areas using mobile technology: A pre- post evaluation of the SMART Mental Health project in rural India	India	MI	238 community, 23 HCW	Education, drama, psychiatric treatment, 8 weeks in community; 3 months with HCW	Intrapersonal Interpersonal Community	Multi-Country Validated Care Access and Stigma Scale at pre- and post- intervention	Significant; CI not given; ES not given
13. Michaels, Corrigan, Buchholz, Brown, Arthur, Netter, MacDonald-Wilson 2014 [11]	Changing stigma through a consumer-based stigma reduction program	US	MI	127 PW MI, 131 HCW	Education, contact, 4 workshops each 2–3 h in duration	Intrapersonal Interpersonal	Multi-Country Validated Scale for MI Stigma at pre- and post-intervention	Significant; CI not given; ES Eta squared 0.08
14. Michalak, Livingston, Maxwell, Hole, Hawke, Parikh 2014 [34]	Using theatre to address mental illness stigma: A knowledge translation study in bipolar disorder	Canada	MI	80 PW MI, 84 HCW	Contact, drama; 50-min performance and 30-min question and answer session	Intrapersonal Community	Multi-Country Validated Scale for MI Stigma at pre-, post-, and 3–4 months after intervention	Significant; CI not given; ES Eta squared 0.11
15. Ngoc, Weiss, Trung 2016 [12]	Effects of the family schizophrenia psychoeducation program for individuals with recent onset schizophrenia in Viet Nam	Vietnam	MI	59 PW MI and caregivers/family	Education; 3, 1.5-h sessions	Intrapersonal Interpersonal	Adapted Validated Scale for MI Stigma at pre- and 6 months post-intervention	Significant; CI not given; ES Eta squared 0.13 and 0.18
16. Orkibi 2014 [35]	The effect of drama-based group therapy on aspects of mental illness stigma	Israel	MI	5 PW MI, 7 community	Drama (therapy); 20 weekly, 2-h sessions	Intrapersonal Community	Multi-Country Validated MI Stigma scales at each of 14 session2	Significant; CI not given; ES not given
17. Patalay, Annis, Sharpe, Newman, Main, Ragunathan, Parkes, Clarke 2017 [17]	A Pre-Post Evaluation of OpenMinds: a Sustainable, Peer-Led Mental Health Literacy Programme in Universities and Secondary Schools	UK	MI	234 community, 40 HCW	Education; 2 workshops over 3 weeks	Interpersonal Community	Social Distance Measure used in the UK at pre-and post-intervention	Significant; CI not given; ES odds ratio: 0.85
18. Pinfold, Thornicroft, Huxley, Farmer 2005 [36]	Active ingredients in anti-stigma programs in mental health	UK	MI	PW MI, community (109 police, 78 adults, 472 school students)	Education, social marketing, contact; 2, 2-h sessions; 2, 50-min school sessions;	Intrapersonal Interpersonal Community	Multi-Country Validated social distance scales at pre- and post-intervention	Significant; CI not given; ES not given
19. Smith Fawzi, Eustache, Oswald, Louis, Surkan, Scanlan, Hook, Mancuso, Mukherjee 2012 [37]	Psychosocial support intervention for HIV-affected families in Haiti: implications for programs and policies for orphans and vulnerable children	Haiti	HIV	168 PLWH, 130 caregivers/family	Social support, coping/counseling of 14 and 15 caregiver support group sessions with and without youth.	Intrapersonal Interpersonal	Multi-Country Validated HIV Stigma Scale at ore- and post-intervention assessment	Significant; CI not given for Stigma Scale; ES not given
20. Snead, Ackerson, Bailey, Schmitt, Madan-Swain, Martin 2004 [38]	Taking charge of epilepsy: the development of a structured psychoeducational group intervention for adolescents with epilepsy and their parents	US	Epilepsy	7 PW epilepsy and caregivers/family	Education; 6-week group intervention	Intrapersonal Interpersonal	Multi-Country Validated Scale for Epilepsy Stigma at pre- and post-intervention	Non-significant; CI not given; ES not given
21. Stuhlmiller 2003 [39]	Breaking down the stigma of mental illness through an adventure camp: A collaborative education initiative	Australia	MI	100 PW MI, 200 community	Education, outdoor adventure over 2 days	Intrapersonal Interpersonal	Unpublished Scale of MI Stigma at pre- and post-intervention	Not significant (no inferential statistics); CI not given; ES not given
22. Thurman, Jarabi, Rice 2012 [40]	Caring for the caregiver: Evaluation of support groups for guardians of orphans and vulnerable children in Kenya	Kenya	Orphans/vulnerable children	766 caregivers/family and 1028 orphans/vulnerable children	Social support; Support groups provided in the community	Intrapersonal Interpersonal	Caregiver marginalization scale used in Rwanda, Validation Information Not Given	Significant; CI not given; ES standardized beta −0.22
23. Uys, Chirwa, Kohi, Greeff, Naidoo, Makoae, Dlamini, Durrheim, Cuca, Holzemer 2009 [41]	Evaluation of a health setting-based stigma intervention in five African countries	5 African countries	HIV	41 PLWH, 177 HCW	Contact, coping/counseling, education in a 2-day workshop	Intrapersonal Community	Multi-Country HIV Stigma Scale for HCW at 3 months pre- and within 1 month post-intervention.	Significant; CI not given; ES not given
24. Yotsumoto, Hirose, Hashimoto 2010 [42]	An awareness program: the significance of lectures delivered by individuals with mental disabilities	Japan	MI	12 PW MI, 844 community	Contact, education; 2–5 lectures per person	Intrapersonal Community	Multi-Country Validated Stigma Scale at post-intervention	Non-significant; CI not given; ES not given

Notes: We categorized findings in terms of statistical significance (at least 1 measure of stigma used showed statistically significant reduction at *p* < 0.05) and non-significance (no statistical significance found or no inferential statistics used). We calculated effect size (ES) when enough information was provided to calculate Cohen’s *d* or Eta squared. Unstandardized test statistics were labeled as ‘ES Not Given’

*Abbreviations*: *CI* confidence interval, *ES* effect size, *HCW* health care workers, *HIC* high-income country, *HIV* human immunodeficiency virus, *LMIC* low- and middle-income country, *MI* mental illness, *PLWH* people living with HIV, *PRISMA* Preferred Reporting Items for Systematic Reviews, *PW* people with, *RCT* randomized controlled trial, *US* United States

We found approximately equal numbers of studies originating from low- and middle-income country (LMIC) and high-income country (HIC) settings, with 13 studies conducted in HIC and 11 studies conducted in LMIC. Five studies were based in the US, three in the UK, two in Canada, two in Indonesia, two in South Africa, and one study spanned five African countries (Lesotho, Malawi, South Africa, Swaziland, and Tanzania). One study was conducted in each of the following countries: Kenya, Zambia, China, India, Vietnam, Israel, Haiti, Australia, and Japan.

Twelve articles examined stigma associated with mental illness, six HIV, two leprosy, one Moebius syndrome, and one each of epilepsy, orphans and vulnerable children, and substance use. Eighteen articles described studies targeting stigmatized participants, 12 included participants who were community members (e.g., students, police), six articles included healthcare workers as participants, eight articles addressed stigma among caregivers/family members, and two articles examined stigma among youth at risk for HIV. Of the articles targeting stigmatized populations, six studies targeted both stigmatized and community populations, eight studies targeted both stigmatized and caregiver populations, and six studies targeted both stigmatized and healthcare worker populations.

Five of the six articles examining HIV-related stigma originated from LMICs. Conversely, articles examining mental illness-related stigma predominantly came from a HIC (e.g., UK, US, Canada), with only one out of 12 articles from a LMIC (India). Five studies were published prior to 2010, whereas 19 were published between 2010 and 2017. Five of the six studies of HIV-related stigma were published after 2010, and nine of 12 studies of mental health-related stigma were published in 2010 or after.

**Fig. 2:**
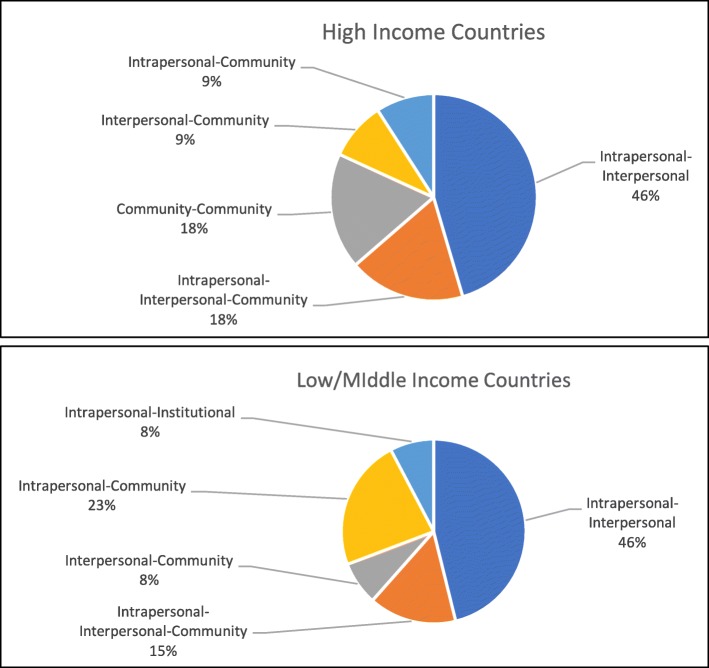
Levels examined together, separated by high- and low/middle-income country

The most common levels examined together were intrapersonal and interpersonal, with 15 articles including these two levels together. Of the 24 articles, four examined the community level alongside both the intrapersonal and interpersonal levels. Three articles examined intrapersonal and community levels together, and another three examined interpersonal and community levels together. Thus, a total of 10 articles examined community levels alongside either interpersonal, intrapersonal, or both levels. Only one article examined the institutional level (along with intrapersonal), and no articles targeted the structural level. Figure 2 depicts these findings, separated by LMIC and HIC study location.

The most common stigma reduction strategy studied was education, with 16 studies using this strategy. Ten studies examined contact, five counseling or coping skills acquisition, three social support, three drama, and two problem solving. Individual studies also examined communication skills, voluntary counseling and testing, psychiatric treatment, and outdoor adventure as stigma reduction techniques. Of the 12 articles that examined the community level alongside at least one other level, six used contact as a primary strategy. Eight studies used education and contact strategies together, and six of these eight studies originated from HIC.

Seven of the 24 studies examined one stigma reduction strategy across more than one level. For example, Patalay and colleagues [17] trained university medical students in the UK to lead workshops with secondary school students on mental health conditions and services (educational strategy). Investigators measured medical students’ levels of stigma and impact on the interpersonal level to examine the workshop leadership’s potential impact on medical students’ future practice behaviors. The researchers also measured attitudes towards mental illness of the recipients of the intervention, (the secondary school students), thereby using the same educational strategy and then assessing community-level stigma. Of note, one study used an educational strategy with specialized areas of content: Brown and colleagues [18] had nursing students deliver a stigma reduction program in the US to community members by providing information on mental health issues as well as on a faith-based framework for dealing with mental health issues. In other words, this intervention provided education on a condition as well as education on using faith to cope with the condition (two educational strategies), without using faith-based counseling techniques as the intervention itself.

The majority (16 of 24) of studies reviewed used stigma scales that were validated and used in multiple countries. The remaining eight studies used scales that were adapted from validated scales, used in a neighboring country, or validated by the study team for use in the country where the study had taken place. Beyond use of validated and adapted measures, the articles provided little information on how well the instruments performed across cultures and contexts.

In terms of effectiveness, 17 studies reported that their intervention reduced stigma scores (*p* < 0.05) on at least one measure of stigma and seven studies reported non-significant results. Of these seven studies that found non-significance, five were conducted in high-income countries and two were conducted in middle-income countries (China and South Africa). Only two of the 24 articles provided information on confidence intervals. In terms of practical significance, only 11 out of the 24 studies provided information to calculate effect sizes or the effect sizes themselves. Cohen’s *d* values that were reported ranged from 0.4 to 2.51, Eta squared and R-squared values ranged from 0.02 to 0.32, indicating small-to-moderate effects across studies.

## Discussion

We set out to review intervention studies that targeted multiple levels of stigma reduction and identified 24 studies. Notably, the majority of studies identified and reviewed were published after 2010, demonstrating an increasing urgency and movement in the research community towards developing and validating stigma reduction interventions. Articles that originated from HICs tended to examine mental illness-related stigma, whereas those from LMICs tended toward the examination of HIV-related stigma. This may be due to availability of funds, as global health spending in LMICs has decreased over time except for HIV-related work [19].

Most investigators used validated or adapted measures of stigma in their studies, but provided little information on how well the measures performed in diverse settings. Contextual psychometric information and sensitivity/specificity of measures are useful pieces of information to determine accurate interpretation of intervention effectiveness. This is particularly relevant for studies that used adapted measures or measures validated in languages or contexts that differed from where the studies were conducted. More detailed examination of measures used to evaluate intervention effectiveness will be an important direction for future research on multi-level interventions.

Similarly, we found relatively few studies that used randomized controlled trial (RCT) designs. The lack of RCT designs may be due to the challenges of conducting RCTs across multiple levels. Investigators in future studies of multi-level stigma interventions may consider use of non-traditional hybrid trial designs, quasi-experimental designs, or other types of pragmatic designs used in complex real-world settings. Similarly, we also noted that just under half of the reviewed articles provided effect sizes, and those that were reported varied widely in magnitude. Adding rigor to these designs may help to narrow information on the potential benefits of interventions that operate on multiple levels.

The intrapersonal and interpersonal levels were most often targeted by the multi-level stigma interventions studied, which may be due to several factors. The broader stigma literature has focused almost exclusively on these two levels of analysis [1]; thus, multi-level interventions have a larger evidence base from which to draw at these levels. Relatedly, research has accumulated a wealth of stigma measures at the individual/interpersonal levels of analysis. In contrast, until recently, fewer measures of stigma existed at community, organizational, and structural levels, which likely hindered the assessment of multi-level stigma interventions that incorporated communities and social structures [20]. This focus on the individual/interpersonal levels in multi-level stigma interventions may also be due to convenience—intervention studies are often easier to implement in clinical settings where people with health-related stigmatizing conditions seek care and where their family members (who are needed for research at the interpersonal level) are more easily identified and assessed. More research is needed to incorporate community-, organizational-, and structural-level influences into multi-level stigma interventions.

Approximately half the studies reviewed examined community-level stigma reduction, with intrapersonal and/or interpersonal levels. Studies that targeted community levels of stigma predominantly used methods of interaction, or contact, across populations studied, examining the impact of exchanging information and making use of bi-directional learning and including people living with stigmatized conditions in the process (e.g., teaching, drama). In addition, these studies tended to incorporate exchanges of support, particularly when family members and health care workers were involved.

Despite accumulating research indicating that structural forms of stigma contribute to adverse health outcomes among members of stigmatized groups [21, 22], only one study combined an institutional-level approach, and no studies combined the structural-level approach, alongside another level. Researchers may consider institutional- and structural-level interventions challenging, since they require time and financial resources to examine stigma in large samples. Despite these challenges, single-level studies are beginning to emerge that examine stigma reduction as a result of policy changes at the structural level [23]. Thus, one important direction for future development of multi-level interventions is greater attention to, and incorporation of, policy-level interventions to address stigma at the institutional and structural levels.

With respect to stigma-reduction strategies used by these multi-level stigma interventions, most focused on education, either alone or in combination with other strategies, such as contact. Corrigan and colleagues found over years of research that stand-alone educational programs can lead to stereotype suppression, in which members of the public suppress—rather than reject—stereotyped beliefs upon learning that such beliefs are socially undesirable [24, 25]. Thus, educational programs alone are often ineffective in reducing stigmatizing attitudes in members of the public, and the little resulting stigma reduction that occurs may be short-lived and superficial [26]. Future research on multi-level stigma interventions is therefore needed to explore a wider range of stigma-reduction strategies and to utilize evidence-based strategies that prior research has shown to be effective in reducing stigma.

This review has several limitations. First, although we introduced independent secondary article reviewers and coders, our process of article selection, non-inclusion of gray literature, inclusion of studies reported in English only, and content analysis may have introduced selection biases that restrict the generalizability of our findings to all multi-level stigma interventions. Second, the scope of our study did not include detailed comments regarding a methodological appraisal of studies and we included limited information on intervention effectiveness. The lack of rigor in these studies may have led to sampling bias and non-generalizable conclusions. Thus, additional research will need to be done before recommendations on effectiveness can be made.

## Conclusions

Stigma is inherently a cross-sectoral phenomenon [1] and thus efforts to reduce stigma and its pernicious effects require a multi-level approach. Despite progress over the past decade in the development of multi-level stigma interventions, much work remains to strengthen and broaden this approach. In Table 2, we highlight several opportunities for new research and program development in multi-level stigma interventions, organized around several key domains (e.g., measurement, mechanisms of change, implementation). This list is not exhaustive, but rather is meant to underscore some of the most important areas of inquiry that are needed to advance the knowledge base in this incipient field. For instance, multi-level stigma interventions may not always be appropriate; future research is therefore needed to systematically compare the efficacy of single-level vs. multi-level stigma interventions in order to determine the conditions under which multi-level stigma interventions may be preferable to single-level interventions. Future research is also needed to evaluate how changes at one level of stigma (e.g., intrapersonal) impact other levels of stigma (e.g., community) in order to guide the development of more effective multi-level interventions, to identify mechanisms of change in multi-level stigma interventions, and to explore the barriers and facilitators to the dissemination of multi-level stigma interventions across diverse contexts. Only after answering these questions will it be possible to fully evaluate whether multi-level stigma interventions are effective in addressing the predicament of stigma in the lives of the stigmatized.

**Table 2:** Future directions for multi-level stigma interventions

Addressing research gaps	
Most multi-level stigma intervention research has focused on the individual/interpersonal level; thus, more research is needed to incorporate community-, organizational-, and structural-level influences into such interventions.	
Most multi-level stigma intervention research has utilized education-based strategies (either alone or in combination with other strategies, like contact) to reduce stigma. Thus, more research is needed across a wider range of stigma-reducing strategies.	
Only a handful of stigmatized groups have been the focus of multi-level stigma reduction interventions, with primary focus on HIV and mental health. Thus, more research is needed to expand the range of groups that are evaluated with these interventions.	
Methods and measurement	
More methodologically rigorous methods are needed to test the efficacy of multi-level stigma interventions, including randomized controlled trials and quasi-experiments.	
New measurement approaches are needed to evaluate synergistic and reciprocal relations of stigma reduction interventions across levels of analysis.	
Multi-level stigma interventions need to more fully engage with several key areas in intervention science, such as implementation science.	
Research questions	
How do changes at one level of stigma (e.g., intrapersonal) impact other levels of stigma (e.g., community)?	
How do multi-level stigma interventions compare to stigma interventions at a single level in terms of efficacy in reducing stigma and/or its negative consequences?	
What are the mechanisms of change? That is, when multi-level stigma interventions are effective, why are they effective?	
How are multi-level stigma interventions that are found effective translated or disseminated? What interpersonal-, community-, and structural-level factors promote or undermine their effective dissemination?	

## Abbreviations

CI: Confidence interval
ES: Effect size
HCW: Health care workers
HIC: High-income country
HIV: Human immunodeficiency virus
LMIC: Low- and middle-income country
MI: Mental illness
PLWH: People living with HIV
PRISMA: Preferred Reporting Items for Systematic Reviews
PW: People with
RCT: Randomized controlled trial
US: United States
